# Problematic facebook use during COVID-19 pandemic among Tunisian women

**DOI:** 10.1192/j.eurpsy.2021.2207

**Published:** 2021-08-13

**Authors:** S. Sediri, Y. Zgueb, A. Aissa, U. Ouali, F. Nacef

**Affiliations:** Psychiatry A Department, Razi Hospital, Manouba, Tunisia

**Keywords:** Addiction, social media, Behavioural addiction, coronavirus

## Abstract

**Introduction:**

Due to COVID-19 pandemic, the Tunisian government officially announced a lockdown on March 2020. This decision caused a significant change in the everyday life of Tunisians such as movement restriction revealing the psychosocial aspect of this crisis.

**Objectives:**

This study aims to assess the impact of the COVID-19 pandemic on the mental health of Tunisian women and its association with social media addiction.

**Methods:**

This study was conducted using an online survey, between April 25 and May 6, 2020. Women were asked about sociodemographic information, lockdown conditions. The Facebook Bergen Addiction Scale (FBAS) was used to evaluate addiction to Facebook and social media. The Depression Anxiety and Stress Scales (DASS-21) was used to evaluate depression, anxiety and stress.

**Results:**

We included 751 participants. Scores of the FBAS ranged from 6 to 30 and the mean score was 16.49 (± 5.4). Forty percent (n = 300) of respondents might have facebook addiction, as per the scale. A significant positive correlation was found between Facebook addiction score and DASS scores of depression (p = 0.001, r = 0.43), anxiety (p = 0.001, r = 0.39) and stress (p = 0.001, r = 0.41).

**Conclusions:**

Women who have higher rates of distress are more likely to have a problematic Facebook use during the COVID-19 pandemic which suggests that smartphone and internet use may be part of coping strategies implemented for the emotional distress secondary to this pandemic.

**Disclosure:**

No significant relationships.

